# Association Between Dietary Total Antioxidant Capacity and Diet Quality in Adults

**DOI:** 10.3389/fnut.2022.838752

**Published:** 2022-04-04

**Authors:** Asma Salari-Moghaddam, Saeedeh Nouri-Majd, Ammar Hassanzadeh Keshteli, Fatemeh Emami, Ahmad Esmaillzadeh, Peyman Adibi

**Affiliations:** ^1^Department of Biochemistry, School of Medicine, Ilam University of Medical Sciences, Ilam, Iran; ^2^Department of Community Nutrition, School of Nutritional Sciences and Dietetics, Tehran University of Medical Sciences, Tehran, Iran; ^3^Department of Medicine, University of Alberta, Edmonton, AB, Canada; ^4^Ebnesina Hospital, Iran University of Medical Sciences, Tehran, Iran; ^5^Department of Community Nutrition, School of Nutrition and Food Science, Isfahan University of Medical Sciences, Isfahan, Iran; ^6^Obesity and Eating Habits Research Center, Endocrinology and Metabolism Molecular-Cellular Sciences Institute, Tehran University of Medical Sciences, Tehran, Iran; ^7^Isfahan Gastroenterology and Hepatology Research Center, Isfahan University of Medical Sciences, Isfahan, Iran

**Keywords:** total antioxidant capacity, diet quality, dietary diversity score, alternate healthy eating index, TAC

## Abstract

**Background:**

Diet quality is a major contributor to human health. In addition, antioxidants have a great contribution to several chronic conditions. The purpose of this study was to evaluate if dietary total antioxidant capacity (TAC) can be considered as a measure of diet quality in a Middle Eastern country.

**Methods:**

In this cross-sectional study on 6,724 Iranian adults, we used a validated food frequency questionnaire (FFQ) to assess dietary intakes. Data derived from the FFQ was used to calculate dietary TAC and well-known diet quality scores including alternate healthy eating index (AHEI) and dietary diversity score (DDS). Dietary TAC was calculated based on the ferric reducing-antioxidant power (FRAP) values reported in earlier publications. AHEI and DDS have also been constructed based on previous publications. Cross-classification was used to examine the agreement between these measures.

**Results:**

Mean age and BMI of study participants were 36.89 ± 8.08 y and 24.97 ± 3.87 kg/m^2^, respectively. We found that individuals in the highest tertile of dietary TAC had higher scores of AHEI (57.53 ± 0.20 vs. 52.03 ± 0.20, *P* < 0.001) and DDS (5.56 ± 0.03 vs. 4.15 ± 0.03, *P* < 0.001) compared with those in the lowest tertile. Participants' distribution on the basis of the cross-classification analysis indicated that the classifications were in exact agreement for 42.6%, within an adjacent tertile for 33.05%, and in gross misclassification for 20% of individuals. When this was examined between dietary TAC and DDS, we found that exact agreement in the classifications was for 59.2% of participants. Notably, a very low proportion of gross misclassification was seen in this regard such that only 6% of participants were classified in the opposing tertiles, indicating additional support for a good agreement.

**Conclusion:**

We found that dietary TAC might be considered as a proper measure for the assessment of diet quality because it was well correlated with well-known measures of diet quality including DDS and AHEI scores.

## Introduction

Diet quality is a major determinant of the increased incidence of chronic diseases (1). It has been well-established that individuals with greater diet quality had a lower risk of cancer (2), diabetes (3) and cardiovascular diseases (4). Although several studies examined diet quality in relation to chronic conditions, the characteristics of a high-quality diet are not well established. Diets with a high quality include high amounts of fruit and vegetables, nuts, fish, legumes, and whole grains (5, 6). The beneficial effects of such diets in disease prevention cannot be attributed to a single nutrient and their effects are likely due to the interactions of all nutrients (7). Antioxidants are among important nutrients in foods included in the high quality diets. Most previous studies examining diet-disease relations have focused on a single antioxidant; however, dietary total antioxidant capacity (TAC) has been developed to assess cumulative, synergic and protective activities of all the antioxidants present in the diet (8). Similar to diets with a high quality, high dietary TAC was also inversely associated with stroke (9, 10), various types of cancer (11–14), cardiovascular diseases (15), diabetes (16), metabolic syndrome (17), and inflammation (18). Therefore, it seems that the effect of high quality diets on disease prevention might be mediated through its high dietary TAC.

Previous studies found a positive association between dietary TAC and dietary quality scores (19, 20). The application of suggested scoring methods for definition of high quality diets is important in the Middle Eastern countries, where the people's dietary intakes have its own characteristics. Given the different nature of diets in different geographical regions along with lack of information in the understudied region of the Middle East, the present study was done to investigate the association between dietary TAC and diet quality in a large sample of Iranian adults.

## Methods and Materials

### Study Design and Population

This cross-sectional study was performed based on data from the Study on the Epidemiology of Psychological, Alimentary Health and Nutrition (SEPAHAN) project, which was a cross-sectional study looking at the prevalence of functional gastrointestinal disorders (FGIDs) and their relationship with lifestyle factors and psychological disorders. Details about SEPAHAN project have been published earlier (21). This study was performed among Iranian general adults working in 50 different healthcare centers affiliated to Isfahan University of Medical Sciences (IUMS) across Isfahan province. To collect information about anthropometric measures, demographic and lifestyle factors, including dietary intakes and physical activity, self-administered questionnaires were distributed among 10,087 subjects, and 8,691 participants returned the completed questionnaires (response rate: 86.16%). In the current analysis, we excluded subjects who reported their total daily energy intake outside the range of 800–4,200 kcal/d. We also excluded those who had missing data on any relevant variable. These exclusions resulted in a dataset of 6,724. All participants provided written inform consent forms. Although the protocol of SEPAHAN study was approved by the Regional Bioethics Committee of Isfahan University of Medical Sciences, the current study was separately approved by the Research Council of School of Nutritional Sciences and Dietetics of Tehran University of Medical Sciences, Tehran, Iran (Ethics code: IR.TUMS.VCR.REC.1398.131).

### Dietary Intakes Assessment

A self-administered, Willett-format, Dish-based, 106- item Semi-quantitative Food Frequency Questionnaire (DS-FFQ), was used to assess dietary intakes. The questionnaire was designed and validated for use in Iranian adults. Details on design, foods included, and the validity of this questionnaire has been reported elsewhere (22). Briefly, we provided a comprehensive list of foods and dishes commonly consumed by Iranian adults. Then, those foods that were nutrient-rich, often consumed, or contributed to between-person variation were selected. Eventually, this process led to remaining of the 106 food items in 5 various categories in the questionnaire: (1) mixed dishes (cooked or canned, 29 items); (2) grains (different types of bread, biscuits, cakes and potato, 10 items); (3) dairy products (dairy, butter and cream, 9 items); (4) fruits and vegetables (22 items); and (5) miscellaneous food items and beverages (including fast foods, nuts, sweets, desserts and beverages, 36 items). In order to provide precise and accurate estimates, the portion size of foods and mixed dishes as a unit with the same perception were given to all people. Nine multiple choice frequency response categories ranging from “never or <1/month” to “≥12/day” were provided for reporting dietary intakes of participants. The number of response categories for the food list varied from 6 to 9 choices. For foods consumed infrequently, we omitted the high-frequency categories, while the number of multiple choice categories increased for common foods with a high intake. Finally, daily intake of all food items was computed and then converted to grams per day using household measures (23). Daily nutrient intakes of each participant were estimated based on the US Department of Agriculture's (USDA) national nutrient databank (24). The validity of DS-FFQ was examined in a subgroup of 200 participants randomly selected for the SEPAHAN project. All participants in the validation study completed the DS-FFQ at study baseline and 6 months later. During this validation study, participants provided three detailed dietary records that were used as the gold standard. As shown in earlier studies (22), it seems that this questionnaire provides reasonably valid measures of long-term dietary intakes. Also, some recent studies have shown that FFQ was a valid questionnaire for assessing dietary quality scores (25).

### Dietary TAC Assessment

Dietary TAC was obtained from previous studies, based on the ferric reducing-antioxidant power (FRAP) values of 100 food items from the 106 selected food items. The food parameters that were not included in the TAC construction included salt, pepper, sugar, sugar loaf, gaz and nabat (traditional Iranian sweets), because the TAC value for these dietary factors was zero. The FRAP assay is a tool measuring the ability of dietary antioxidants to reduce ferric to ferrous ions. The FRAP values express as mmol per 100 grams of foods (mmol/100 g) (26). For similar food items in Iranian culture (e.g., several types of breads), we calculated the overall mean value. Finally, the frequencies of consumption of each food item were multiplied by their related FRAP values and then summed up to obtain dietary TAC for each participant.

### Dietary Diversity Score

A method described by Kant et al. (27, 28) was used for scoring dietary diversity. This method was based on five groups including grains, vegetables, fruits, meats, and dairy, all food groups in the USDA food guide pyramid. The grains group was composed of seven components: refined bread, macaroni, whole grain bread, corn flakes, rice, biscuit, and refined flour. As we had no data about intake of corn flakes, we decided to consider six components. Fruit was defined by summing up fruit and fruit juice, berries, and citrus fruits. In terms of vegetables, we summed up mixed vegetables, potato, tomato, other starchy vegetables, legumes, yellow vegetables, and green vegetables. The group of meat was composed of red meat, poultry, fish, and eggs and the group of dairy was composed of milk, yogurt, and cheese.

Participants were considered as a “consumer,” and scored as 1, for each component of food groups if they had intakes higher than median levels; otherwise they were given the score of 0. Then the scores for components in each food group were summed up to have total score of that food group. Then, we divided total scores obtained in each group to the number of components in that group. This value was then multiplied by 2. Total DDS for each participant was then computed by summing up the figures for food groups. For example, in the grains group, if a person had dietary intakes of whole grain bread, macaroni, and rice higher than the median values, her or his score was calculated as (3/6) × 2 = 1. Therefore, the diversity score for the grains group would be 1 for that person. After computing the diversity score for the other four groups in that person, total DDS was computed. Therefore, minimum and maximum scores of total dietary diversity for each participant were between 0 and 10.

### Alternate Healthy Eating Index

To calculate AHEI-2010, a method designed by Kennedy et al. was used (29–31). AHEI-2010 consisted of eleven components: fruit, vegetables, whole grains, nuts and legumes, long-chain n-3 fats (DHA and EPA), PUFA, alcohol consumption, sugar-sweetened drinks and fruit juice, red and processed meats, trans-fat and sodium. In the current study, alcohol consumption was not included into the score, because of the lack of information in the original dataset. To construct the index, first we obtained energy-adjusted intakes of the above-mentioned components by using the residual method (32). Next, participants were classified based on decile categories of energy-adjusted intakes of these components. As scoring by deciles would be least prone to misclassification, we used decile categories of components instead of other classifications. Individuals in the highest deciles of fruits, vegetables, whole grains, nuts and legumes, long-chain n-3 fats and PUFA were given a score of 10, and those in the lowest decile of these items were given a score of 1. Individuals in the other deciles of these components were assigned the corresponding scores. Regarding sugar-sweetened drinks and fruit juice, red and processed meat, trans-fatty acids, and sodium intake, the lowest decile was given a score of 10 and the highest decile was given a score of 1. Those in deciles 9, 8, 7, 6, 5, 4, 3, and 2 of these components were given scores of 2, 3, 4, 5, 6, 7, 8, and 9, respectively. The whole AHEI-2010 was computed through summing up the scores of its components ranging from 10 to 100.

### Assessment of Other Variables

Required information on other variables including age, sex, marital status, smoking status, and education was obtained from demographic and medical history questionnaires. Physical activity was assessed using the General Practice Physical Activity Questionnaire (GPPAQ) (18). Based on participants' responses, they were classified into 4 categories; (1) inactive, (2) moderately inactive, (3) moderately active, (4) active. However, in the current study, due to low number of subjects in some of the above-mentioned categories, individuals in the “inactive” and “moderately inactive” groups were combined and were defined as those with “sedentary physical activity”. Similarly, individuals in the “moderately active” and “active” categories were combined and then defined as “physically active”. Anthropometric measures including weight and height were assessed using a self-administered questionnaire. Body Mass Index (BMI) was calculated by dividing weight (kg) to height (m^2^). The correlation coefficient for computed BMI from self-reported values, and the one from measured values was 0.70 (*P* < 0.001).

### Statistical Analysis

We classified participants based on tertiles cut-off points of dietary TAC. General characteristics of study participants across tertiles of dietary TAC were presented as means ± SDs for continuous variables and percentages for categorical variables. To examine the differences across tertiles, we used ANOVA for continuous variables and chi-square test for categorical variables. The multivariable-adjusted means for AHEI and DDS across tertiles of dietary TAC were computed and compared using ANCOVA. In these analyses, energy intake was controlled for in the first model. Further adjustments were made for age (continuous) and sex (male/female) in the second model. BMI (continuous) was controlled for in the third model. Cross-classification of participants across tertiles of TAC, DDS and AHEI was examined. In this analysis, exact agreement was defined when individuals were classified in the same tertiles based on TAC and DDS or AHEI. When individuals were classified in the opposing tertiles, this was considered as gross misclassification. All statistical analyses were done using the Statistical Package for Social Sciences (version 20; SPSS Inc.). *P* < 0.05 was considered as statistically significant.

## Results

Median and range of TAC, DDS, and AHEI was 2010.68 (range: 418.09–5,247.56), 4.92 (range: 0–10), and 55 (range: 26–90), respectively. General characteristics of study participants across tertiles of dietary TAC are shown in Table 1. Participants in the top tertile of dietary TAC were more likely to be older, physically active, current smokers, and university graduated and less likely to be female. No significant difference was found in terms of other variables.

**Table 1:** General characteristics of study participants across tertiles of dietary TAC.

	**Tertiles of dietary TAC**			
	**T_**1**_**	**T_**2**_**	**T_**3**_**	***P*-value^**a**^**
	**(418.1–1,720.4)**	**(1,720.5–2,332.4)**	**(2,333.2–5,247.5)**	
Age, y	36.48 ± 8.05	36.61 ± 7.87	37.58 ± 8.29	<0.001
BMI, kg/m^2^	24.92 ± 3.93	24.88 ± 3.80	25.10 ± 3.86	0.12
Energy intake, kcal/d	1,616.5 ± 473	2,375.5 ± 572	3,122.9 ± 631	<0.001
Female, %	62.5 (1,401)	59.7 (1,339)	54.8 (1,229)	<0.001
Married, %	83.3 (1,820)	82.8 (1,816)	82.5 (1,811)	0.46
Physically active (≥1 h/week), %	32.5 (663)	32.4 (675)	36.0 (752)	0.02
Overweight or obese, %	45.6 (1,018)	45.9 (1,026)	48.6 (1,083)	0.08
Current smokers, %	3.0 (58)	3.3 (64)	5.3 (105)	<0.001
Education (university graduate), %	56.3 (1,234)	63.5 (1,393)	60.8 (1,330)	<0.001

*Data are mean ± standard deviation (SD) or percent (number)*.

a *Obtained from ANOVA or chi-square test, where appropriate*.

*TAC, total antioxidant capacity*.

Crude and multivariable-adjusted means for AHEI and DDS across tertiles of dietary TAC are shown in Table 2. After controlling for energy intake, age, sex, and BMI, we found that individuals in the highest tertile of dietary TAC had higher scores of AHEI compared with those in the lowest tertile (57.53 ± 0.20 vs. 52.03 ± 0.20, *P* < 0.001). In the fully adjusted model, individuals in the top tertile of dietary TAC had higher scores of DDS (5.56 ± 0.03 vs. 4.15 ± 0.03, *P* < 0.001) compared with those in the bottom tertile.

**Table 2:** Mean scores of AHEI and DDS across tertiles of dietary TAC.

	**Tertiles of dietary TAC**			
	**T_**1**_**	**T_**2**_**	**T_**3**_**	***P*-value^**a**^**
**Subjects, *n***	**2,241**	**2,242**	**2,241**	
**AHEI**				
Crude	52.06 ± 0.16	54.87 ± 0.16	57.52 ± 0.16	<0.001
Model I	51.99 ± 0.20	54.87 ± 0.16	57.58 ± 0.20	<0.001
Model II	52.00 ± 0.20	54.87 ± 0.16	57.57 ± 0.20	<0.001
Model III	52.03 ± 0.20	54.87 ± 0.16	57.53 ± 0.20	<0.001
**DDS**				
Crude	3.33 ± 0.03	5.03 ± 0.03	6.37 ± 0.03	<0.001
Model I	4.15 ± 0.03	5.03 ± 0.02	5.56 ± 0.03	<0.001
Model II	4.15 ± 0.03	5.03 ± 0.02	5.55 ± 0.03	<0.001
Model III	4.15 ± 0.03	5.03 ± 0.02	5.56 ± 0.03	<0.001

*Data are mean ± standard error (SE)*.

a *Obtained from ANCOVA*.

*Model I: adjusted for energy intake*.

*Model II: additionally, adjusted for age and sex*.

*Model III: additionally, adjusted for BMI*.

*AHEI, alternate healthy eating index; DDS, dietary diversity score; TAC, total antioxidant capacity*.

Participants' distribution on the basis of cross-classification analysis between tertiles of dietary TAC and AHEI indicated that the classifications were in exact agreement for 42.6%, within an adjacent tertile for 33.05%, and in gross misclassification for 20% of individuals. When this was examined between dietary TAC and DDS, we found that exact agreement in the classifications was for 59.2% of participants. Notably, a very low proportion of gross misclassification was seen in this regard such that only 6% of participants were classified in the opposing tertiles, indicating additional support for a good agreement (Table 3).

**Table 3:** Participants' distribution across tertiles of dietary TAC, AHEI, and DDS.

	**Tertiles of dietary TAC**			
	**T_**1**_**	**T_**2**_**	**T_**3**_**	***P*-value^**a**^**
**AHEI**				<0.001
T_1_	47.7 (1,070)	33.6 (753)	21.4 (479)	
T_2_	33.7 (755)	34.3 (769)	32.8 (735)	
T_3_	18.6 (416)	32.1 (719)	45.8 (1,027)	
**DDS**				<0.001
T_1_	68.1 (1,525)	24.7 (553)	7.7 (173)	
T_2_	27.6 (619)	44.3 (993)	27.0 (605)	
T_3_	4.3 (97)	31.0 (696)	65.3 (1,463)	

*Data are percent (number)*.

a *Obtained from chi-square test*.

*AHEI, alternate healthy eating index; DDS, dietary diversity score; TAC, total antioxidant capacity*.

## Discussion

In this cross-sectional study, we investigated if dietary TAC can be considered as a measure for healthy eating. We found that participants in the highest tertiles of dietary TAC had higher scores of AHEI and DDS as well. Given the proper agreements between dietary TAC and AHEI and DDS, we concluded that dietary TAC can be a good and appropriate measure for healthy eating.

Dietary TAC was used to assess dietary antioxidants. It was associated with reduced risk of various chronic diseases (9, 10, 14–17). On the other hand, indicators of healthy eating were also previously linked with a lower risk of mortality and several chronic conditions. Therefore, we assumed that dietary TAC might be a good indicator for diet quality as well. Poor diet quality has been associated with increased risk of chronic diseases (33). Identification of nutrients involved in increasing the quality of the diet might help individuals to make accurate food choices. Investigating the association between dietary TAC and diet quality is a novel concept and few studies have been done in this regard so far. We found that high quality diets contained greater dietary TAC. In line with our study, Ha et al. reported that high dietary TAC was correlated with greater adherence to the diet quality index scores (DQIS) including Healthy Eating Index (HEI), Alternative Healthy Eating Index (AHEI), alternate Mediterranean Diet (aMED), and Dietary Approaches to Stop Hypertension (DASH) (20). Similar findings were also reported by Puchau et al. in which dietary TAC was positively associated with dietary quality scores including HEI, AHEI, Diet Quality Index-International (DQII), Diet Quality Index-Revised (DQIR), Mediterranean Diet Score (MDS), Alternate Mediterranean Diet Score (AMDS), Modified Mediterranean Diet Score (MMDS), Quantitative Index for Dietary Diversity (QIDD), and Recommended Food Score (RFS) (19). Overall, it seems that dietary TAC can be considered as a measure of diet quality. In other words, recommending general population to increase their dietary antioxidants intake might result in increased quality of their diet, as measured by indicators of diet quality.

An imbalance between antioxidants and pro-oxidants in the body leads to oxidative stress, which is the basis for several diseases. Oxidative stress occurs when there is an overproduction of ROS or an enzymatic or non-enzymatic deficiency of antioxidants in the body. Fruits and vegetables as rich sources of antioxidants in the diet can act as anti-aging agents and are beneficial for health (34). Greater dietary TAC, as compared with a low dietary TAC, has been associated with lower levels of inflammation, as measured by high-sensitivity C-reactive protein (35). In addition, dietary TAC was positively associated with food group intake including fruits, vegetables, whole grains, legumes, nuts, seeds, and seafood, and inversely related to red and processed meat consumption (20). Therefore, it seems that dietary TAC is a good measure to classify food groups in terms of their contribution to human health and may also be considered to measure the quality of the diet. Application of TAC assessment might be that, instead of the whole diet assessment, we can assess limited numbers of dietary factors to assess peoples' adherence to a healthy diet.

The use of cross-classification of participants in terms of dietary TAC and AHEI and large sample size of the study might be considered as some strengths of this study. In addition, it must be kept in mind that these findings came from a region where data on dietary information and dietary measurements by valid methods are scarce. Given the unique characteristics of diet in Middle Eastern countries, having information about appropriate measures to define quality diets in this area is of importance. However, this study has some limitations that should be considered when interpreting our results. Due to the application of FFQ for dietary assessment, measurement errors and misclassification of participants is unavoidable. However, we used a validated FFQ for assessment of dietary intakes to minimize the bias in dietary assessments. In addition, we did not examine the validity of this questionnaire for assessment of dietary TAC. However, earlier publications based on this questionnaire have revealed that data on dietary TAC from this questionnaire can be used to predict the chronic conditions (36). Moreover, some components of DDS and AHEI including corn flakes and alcohol consumption were not considered in this study, because of the lack of data in the original data set. The usage of FRAP to assess the TAC of the food items is another limitation. This essay has an inherent error where the reagents react with atmospheric Oxygen, thereby, rendering the values interfered.

In conclusion, we found that dietary TAC might be considered as a proper measure for assessment of diet quality, because it was well correlated with well-known measures of diet quality including DDS and AHEI scores.

## Data Availability Statement

The raw data supporting the conclusions of this article will be made available by the authors, via email request to the corresponding author.

## Ethics Statement

The studies involving human participants were reviewed and approved by Regional Bioethics Committee of Isfahan University of Medical Sciences. The patients/participants provided their written informed consent to participate in this study.

## Author Contributions

AS-M, SN-M, FE, and AK: conceptualization, formal analysis, writing—original draft, and writing—review and editing. AE: supervision, conceptualization, methodology, investigation, funding acquisition, formal analysis, writing—original draft, and writing—review and editing. PA: conceptualization, investigation, and methodology. All authors contributed to the article and approved the submitted version.

## Conflict of Interest

The authors declare that the research was conducted in the absence of any commercial or financial relationships that could be construed as a potential conflict of interest.

## Publisher's Note

All claims expressed in this article are solely those of the authors and do not necessarily represent those of their affiliated organizations, or those of the publisher, the editors and the reviewers. Any product that may be evaluated in this article, or claim that may be made by its manufacturer, is not guaranteed or endorsed by the publisher.
